# Supplementary material for: Phycobilisome light-harvesting efficiency in natural populations of the marine cyanobacteria Synechococcus increases with depth
Source: Commun Biol. 2022 Jul 22;5:727. doi: 10.1038/s42003-022-03677-2 (PMC9307576; doi:10.1038/s42003-022-03677-2)
Supplement: Supplementary file 2 — Supplementary Information [file 42003_2022_3677_MOESM2_ESM.pdf]

Supplementary information

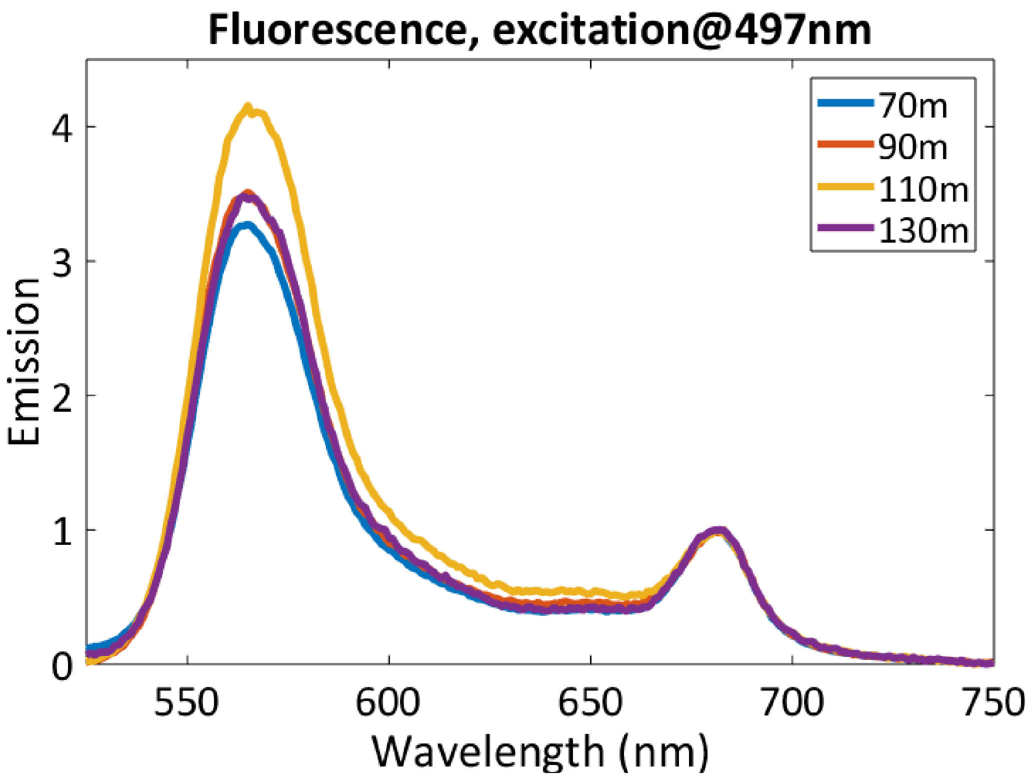

Supplementary Figure 1. Steady-state fluorescence spectra example of the measured seawater samples. Although the samples contain additional organisms other than *Synechococcus*, the excitation at 497nm, directed at phycoerythrin, leads to significantly higher fluorescence intensity of phycobilisomes (540-640 nm) than of photosystems. Still, the observed fluorescence of PSII (670-700 nm) is composed both from fluorescence of *synechococcus* and other organisms.

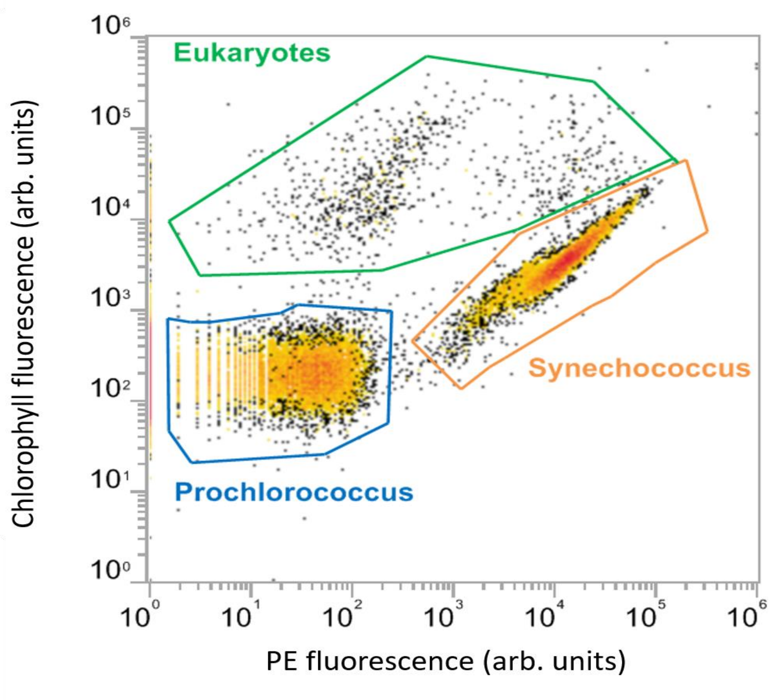

Supplementary Figure 2. Flow cytometry gating of *Synechococcus* cells based on fluorescence properties. *Synechococcus* cells were detected according to levels of PE and chlorophyll fluorescence (arb. units) following protocol by Marie et al. 1997.

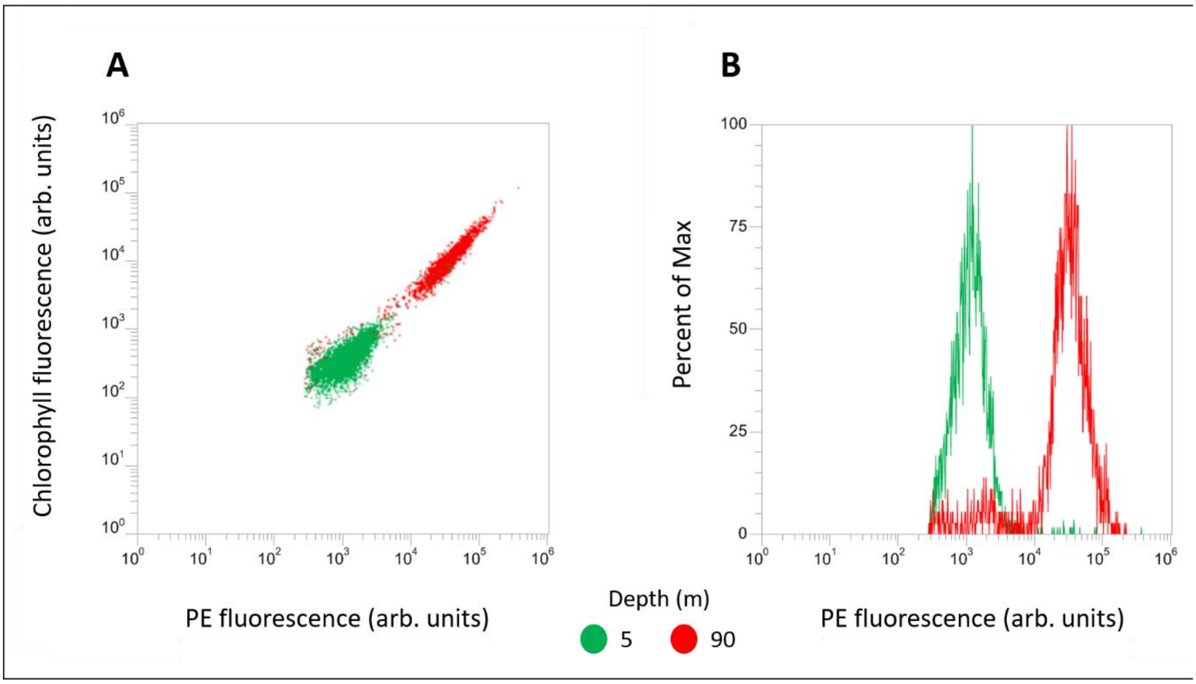

Supplementary Figure 3. Flow cytometry plots of *Synechococcus* cells at different depths during summer stratification (August 2020). *Synechococcus* cells were detected according to levels of PE and chlorophyll fluorescence following protocol by Marie et al. 1997 (A). Histogram of PE fluorescence measured for *Synechococcus* in different depths. Cells found at surface depth are shown in green, cells found at the DCM are shown in red (B).

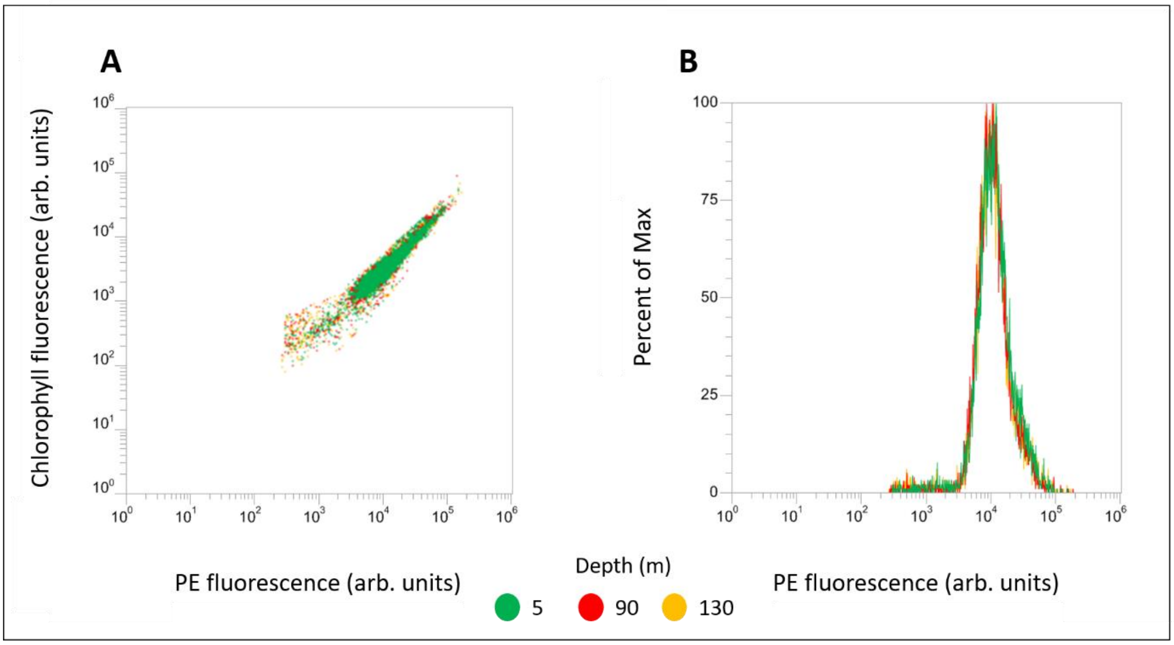

Supplementary Figure 4. Flow cytometry plots of *Synechococcus* cells at different depths during winter mixing (February 2021). *Synechococcus* cells were detected according to levels of PE and chlorophyll fluorescence following protocol by Marie et al. 1997 (A). Histogram of PE fluorescence measured for *Synechococcus* in different depths.

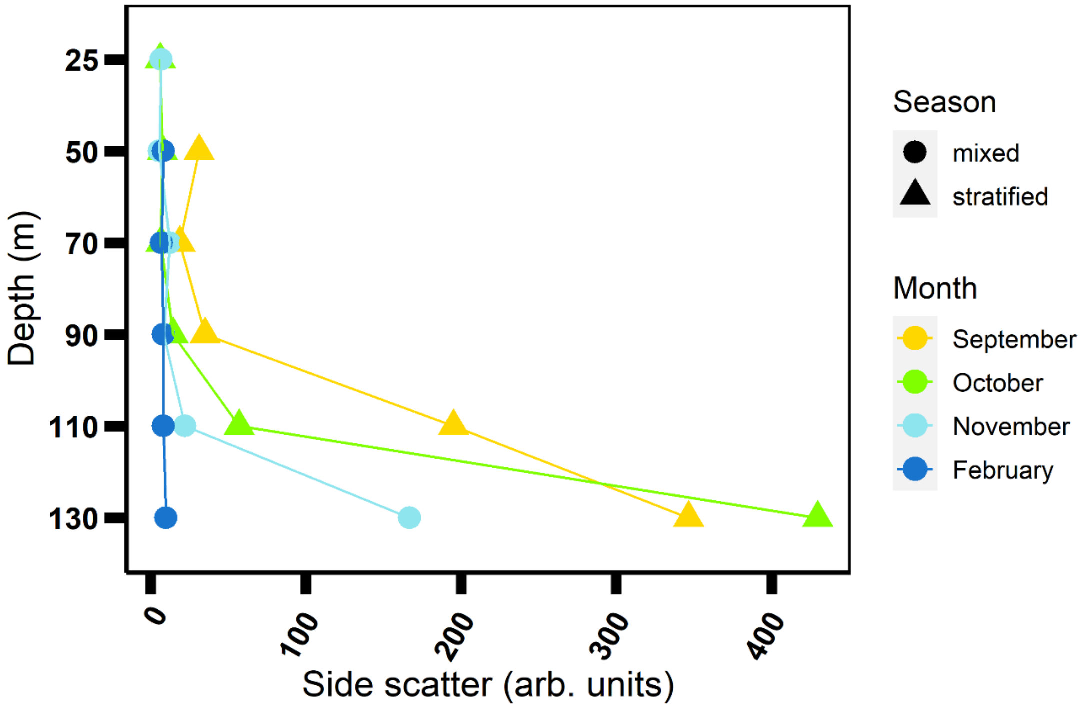

Supplementary Figure 5. Side scattering (SSC) of *Synechococcus* cells, as a function of depth and season, measured by Flow Cytometry. When the water column was stratified (September-October), cells at the deeper layers (below 100 m) were larger.

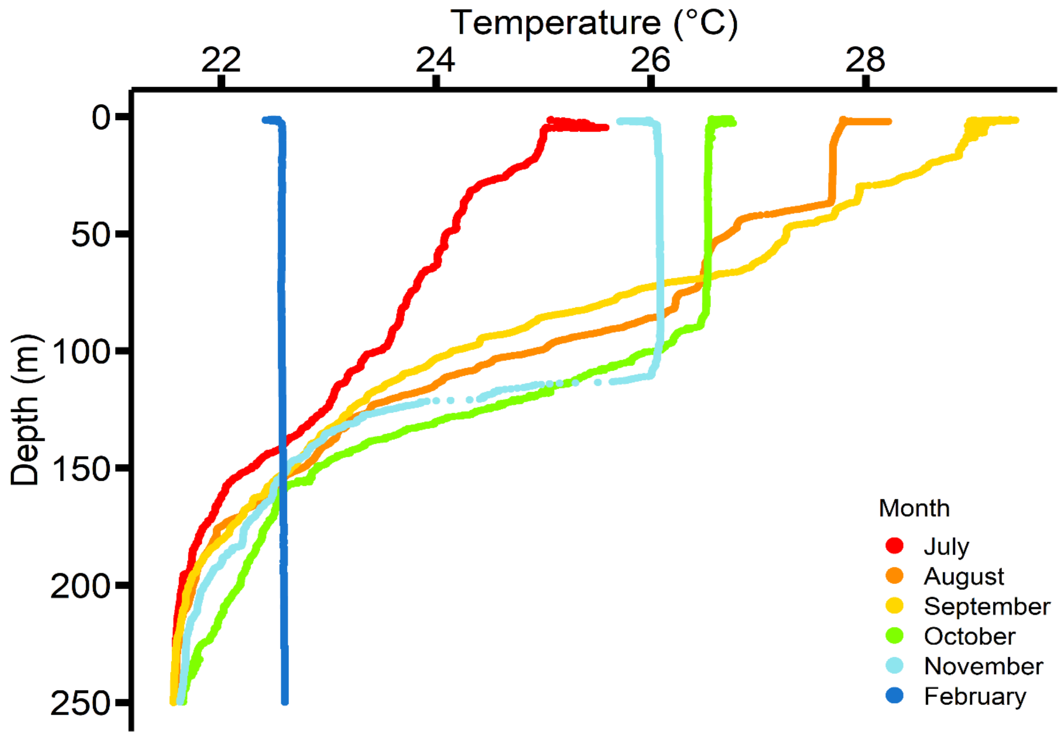

Supplementary Figure 6. Temperature profiles of the water column, measured using a CTD during the sampling cruises, and from additional National Monitoring Program cruises in the same location (Station A., Gulf of Aqaba).

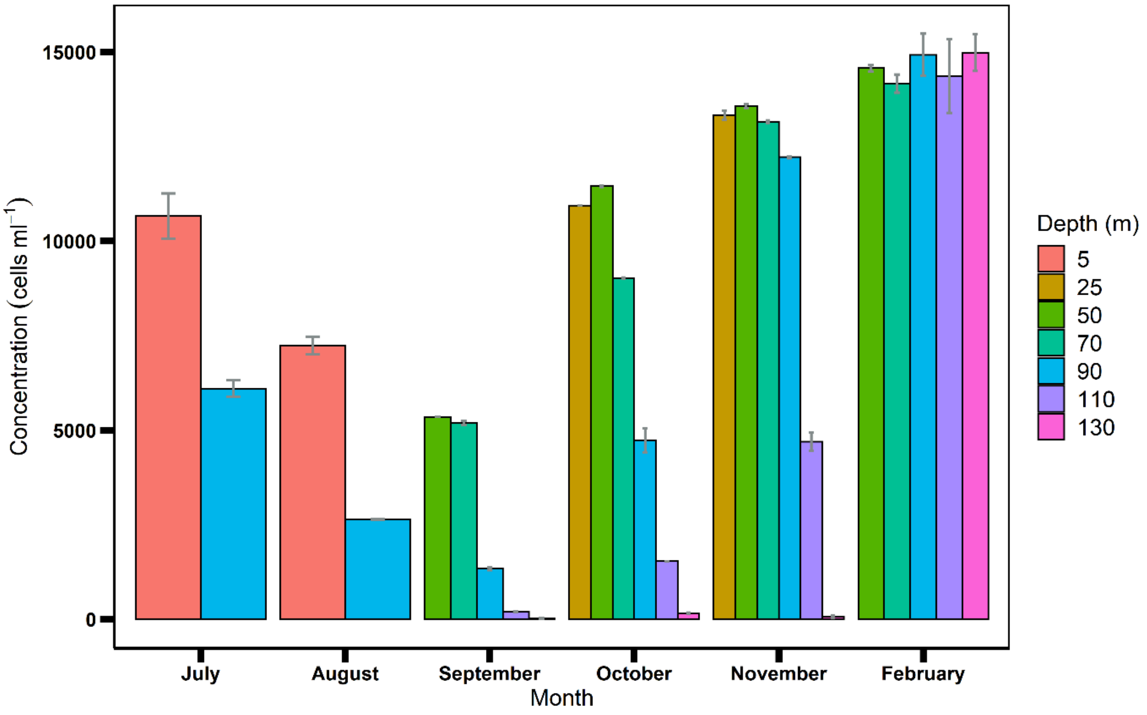

Supplementary Figure 7. Cell concentration of *Synechococcus* during summer and winter. Each color is assigned to a different depth. Error bars represent standard deviation, based on three biological repeats.

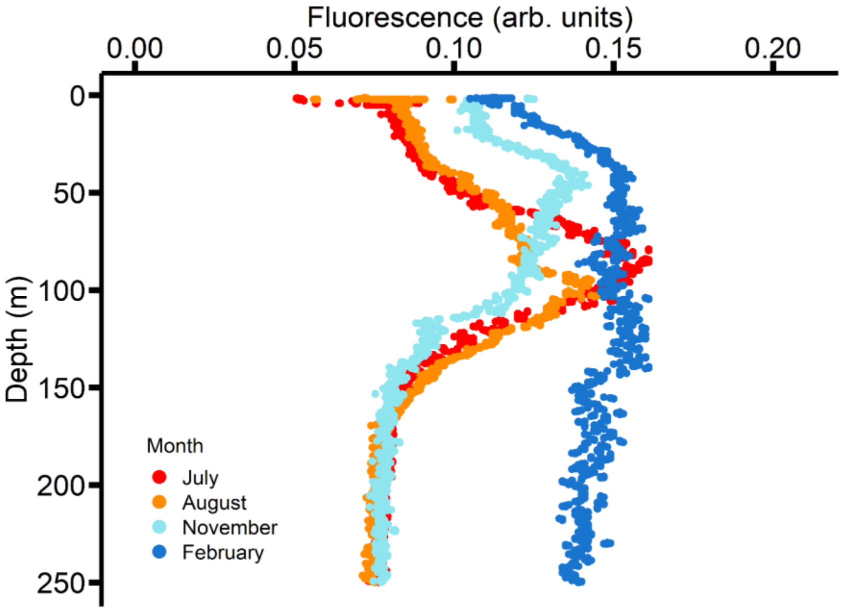

Supplementary Figure 8. Chlorophyll fluorescence profiles of the water column, measured using CTD during the sampling cruises where fluorescence lifetime was measured.

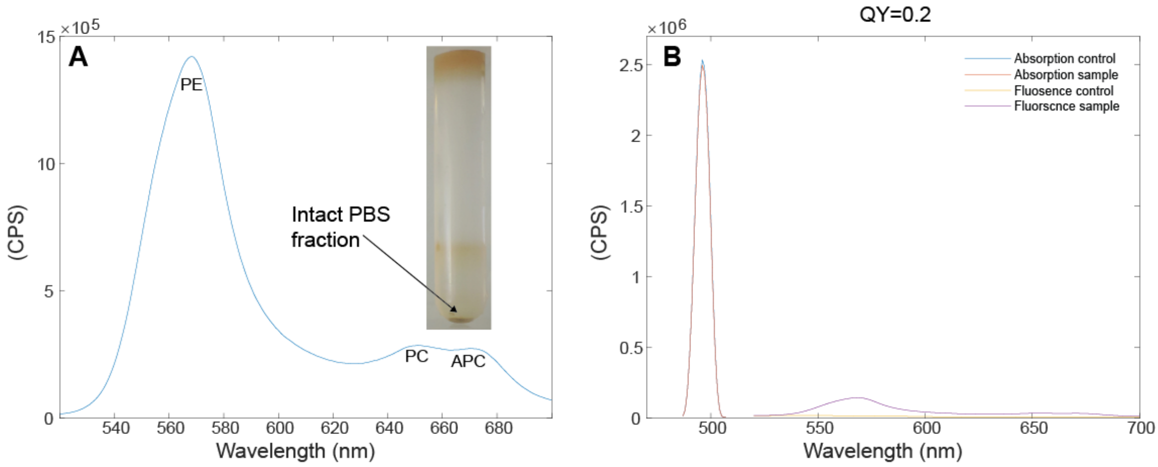

Supplementary Figure 9. Natural lifetime of PBS fluorescence. PBS were isolated from *Synechococcus WH8102* cultures as described by Kolodny and coworkers (2020). Briefly, cells were broken by French press and the thylakoid member fraction was removed by ultracentrifugation. The soluble supernatant containing PBS was loaded on a step sucrose gradient. Three fractions were observed in the gradient (A) top, middle and bottom. The bottom fraction presented the most intact PBS fluorescence spectra including PE, PC and APC peaks (A). This fraction was used for QY quantification in an integrating sphere fitted fluorimeter (B) and lifetime measurement using TCSPC. Measurements were performed on control 0.8M phosphate buffer and on PBS from the bottom fraction diluted 200-fold into the buffer, to ensure that the sample is optically thin. The absorption cross-section, at the excitation wavelength, was calculated from the difference between control and sample measurements around 497 nm. Fluorescence was measured between 520-700 nm. The ratio of fluorescence to absorption, after correction to the detector sensitivity and excitation beam intensity, yielded a QY value of 0.2. The average lifetime of the intact PBS fraction was 2.01-2.28 ns. Using this data we could calculate an apparent natural lifetime value (τ_n) for an intact PBS - τ_n = 10-11.5 ns.

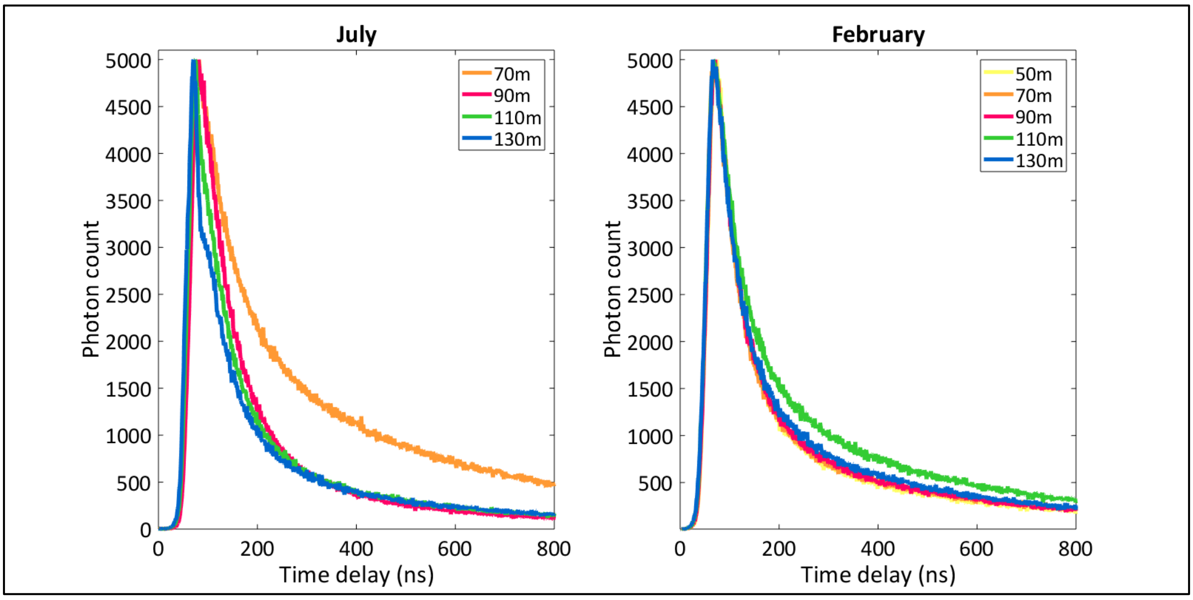

Supplementary Figure 10. Fluorescence lifetime measurements performed using TCSPC technique. Colored lines represent histograms of the number of counted photons (by a single photon detector) at different delay times from the laser's excitation beam, in nanoseconds. Average decay times are calculated from these histograms by performing deconvolution with the system's IRF, and fitting the result to a multi-exponential decay model, as explained in the methods.
