# Supplementary material for: Phycobilisome light-harvesting efficiency in natural populations of the marine cyanobacteria Synechococcus increases with depth
Source: Commun Biol. 2022 Jul 22;5:727. doi: 10.1038/s42003-022-03677-2 (PMC9307576; doi:10.1038/s42003-022-03677-2)
Supplement: Supplementary file 3 — Description of Additional Supplementary Files [file 42003_2022_3677_MOESM3_ESM.pdf]

Description of Additional Supplementary Files

File name: Supplementary Data 1
Description: The source data for Flow Cytometry, CTD, and TCSPC.
