# Supplementary material for: Phycobilisome light-harvesting efficiency in natural populations of the marine cyanobacteria Synechococcus increases with depth
Source: Commun Biol. 2022 Jul 22;5:727. doi: 10.1038/s42003-022-03677-2 (PMC9307576; doi:10.1038/s42003-022-03677-2)
Supplement: Supplementary file 5 — Reporting Summary [file 42003_2022_3677_MOESM5_ESM.pdf]

nature portfolio

Corresponding author(s): Nir Keren
Last updated by author(s): Jun 2, 2022

Reporting Summary

Nature Portfolio wishes to improve the reproducibility of the work that we publish. This form provides structure for consistency and transparency in reporting. For further information on Nature Portfolio policies, see our Editorial Policies and the Editorial Policy Checklist.

Statistics

For all statistical analyses, confirm that the following items are present in the figure legend, table legend, main text, or Methods section.

n/a	Confirmed	
☒	☐	The exact sample size (*n*) for each experimental group/condition, given as a discrete number and unit of measurement
☒	☐	A statement on whether measurements were taken from distinct samples or whether the same sample was measured repeatedly
☒	☐	The statistical test(s) used AND whether they are one- or two-sided<br>*Only common tests should be described solely by name; describe more complex techniques in the Methods section.*
☒	☐	A description of all covariates tested
☒	☐	A description of any assumptions or corrections, such as tests of normality and adjustment for multiple comparisons
☒	☐	A full description of the statistical parameters including central tendency (e.g. means) or other basic estimates (e.g. regression coefficient) AND variation (e.g. standard deviation) or associated estimates of uncertainty (e.g. confidence intervals)
☒	☐	For null hypothesis testing, the test statistic (e.g. *F*, *t*, *r*) with confidence intervals, effect sizes, degrees of freedom and *P* value noted<br>*Give P values as exact values whenever suitable.*
☒	☐	For Bayesian analysis, information on the choice of priors and Markov chain Monte Carlo settings
☒	☐	For hierarchical and complex designs, identification of the appropriate level for tests and full reporting of outcomes
☒	☐	Estimates of effect sizes (e.g. Cohen's *d*, Pearson's *r*), indicating how they were calculated

Our web collection on statistics for biologists contains articles on many of the points above.

Software and code

Policy information about availability of computer code

Data collection	Attune NxT Software version 3.2.1<br>Microsoft Excel version 365 ProPlus
Data analysis	Fluorescence Decay Analysis Software 1.4<br>Rstudio version 4.0.5<br>ggplot2 package 3.3.5<br>Rmisc package 1.5

For manuscripts utilizing custom algorithms or software that are central to the research but not yet described in published literature, software must be made available to editors and reviewers. We strongly encourage code deposition in a community repository (e.g. GitHub). See the Nature Portfolio guidelines for submitting code & software for further information.

Data

Policy information about availability of data

All manuscripts must include a data availability statement. This statement should provide the following information, where applicable:

- Accession codes, unique identifiers, or web links for publicly available datasets
- A description of any restrictions on data availability
- For clinical datasets or third party data, please ensure that the statement adheres to our policy

Relevant data are available from the authors upon request.

Field-specific reporting

Please select the one below that is the best fit for your research. If you are not sure, read the appropriate sections before making your selection.

☐ Life sciences ☐ Behavioural & social sciences ☒ Ecological, evolutionary & environmental sciences

For a reference copy of the document with all sections, see nature.com/documents/nr-reporting-summary-flat.pdf

Ecological, evolutionary & environmental sciences study design

All studies must disclose on these points even when the disclosure is negative.

Study description	Seasonal depth profile analysis of light-harvesting efficiency focused on natural communities of marine cyanobacteria Synechococcus.
Research sample	Samples consisted of marine cyanobacteria Synechococcus found in seawater, collected on different seasons at discrete depths (25-130m).
Sampling strategy	At each cruise, 5L of seawater from different depths was collected by 12L Niskin bottles mounted on a submersible rosette. This volume was filtered for fluorescence life time measurements. Additional volume of seawater was collected and fixed for flow cytometry. Sample size was quantified by flow cytometry. Minimal sample size was recorded at 130m (November 2020), n=94 events within Synechococcus gating.
Data collection	Fluorescence lifetime was determined by using Time Correlated Single Photon Counting (TCSPC) technique. Concentration and cell properties were analyzed with Attune NxT flow cytometer.
Timing and spatial scale	Sampling took place between July 2020- February 2021 to capture seasonal variations of the water column. At each season (summer and winter), two cruises took place as part of Israel National Monitoring Program which takes place once a month. Niskin bottles were filled during one cast at depths ranging from 25-130m.
Data exclusions	Data were not excluded from this study.
Reproducibility	The data and methods are presented to ensure that others will be able to reproduce our results.
Randomization	Randomization was not used, analyses were performed on the entire Synechococcus community found by each seawater collection.
Blinding	Blinding was not relevant to the study, as we did not include application of treatments.

Did the study involve field work? ☒ Yes ☐ No

Field work, collection and transport

Field conditions	Station A is located in the Gulf of Aqaba, in open water away from a continental shelf, where bottom depth is greater than 750 m. The water is characterized by high salinity (40 PSU) with temperature ranging between 21-28 C, and prevalence of North-East winds.
Location	29.5° N; 34.95° E
Access & import/export	Sampling took place as part of the Israel National Monitoring Program's monthly cruises. Sampling was performed on board of the Sam Rothberg RV, where usual cruise duration was 5 hours.
Disturbance	In the study we joined ongoing sampling campaign, thus minimizing working hours and pollution by the boat activity.

Reporting for specific materials, systems and methods

We require information from authors about some types of materials, experimental systems and methods used in many studies. Here, indicate whether each material, system or method listed is relevant to your study. If you are not sure if a list item applies to your research, read the appropriate section before selecting a response.

Materials & experimental systems

n/a	Involved in the study	
☒	☐	Antibodies
☒	☐	Eukaryotic cell lines
☒	☐	Palaeontology and archaeology
☐	☒	Animals and other organisms
☒	☐	Human research participants
☒	☐	Clinical data
☒	☐	Dual use research of concern

Methods

n/a	Involved in the study	
☒	☐	ChIP-seq
☐	☒	Flow cytometry
☒	☐	MRI-based neuroimaging

Animals and other organisms

Policy information about studies involving animals; ARRIVE guidelines recommended for reporting animal research

Laboratory animals	The study did not involve laboratory animals.
Wild animals	The study did not involve wild animals.
Field-collected samples	Field-collected seawater containing microorganisms was fixed upon collection. Fixed samples were kept frozen (-80 C) prior to analysis.
Ethics oversight	No ethical approval or guidance was required, sampling was limited to collection of seawater.

Note that full information on the approval of the study protocol must also be provided in the manuscript.

Flow Cytometry

Plots

Confirm that:

☒ The axis labels state the marker and fluorochrome used (e.g. CD4-FITC).

☒ The axis scales are clearly visible. Include numbers along axes only for bottom left plot of group (a 'group' is an analysis of identical markers).

☒ All plots are contour plots with outliers or pseudocolor plots.

☒ A numerical value for number of cells or percentage (with statistics) is provided.

Methodology

Sample preparation	Duplicates of 4 mL from each depth and additional 4 mL blank (0.22 µm filtered-seawater) for total phytoplankton counts were fixed with 0.25% Glutaraldehyde and 0.01% poloxamer. Tubes were incubated for 30 min in dark, 4°C, followed by flash freeze with liquid N2, and stored in -80°C. Samples were thawed upon analysis and mixed gently.
Instrument	Attune NxT flow cytometer
Software	Attune NxT Software version 3.2.1
Cell population abundance	Cell population was compared with blank measurements. Cells detected in blank sample were subtracted from final population abundance.
Gating strategy	Samples were analysed with 488 nm laser excitation for 5 min, at a rate of 100 µL min-1. Emission was examined at 574/26 to detect orange fluorescence of PE, and 695/40 to detect red fluorescence of chlorophyll a. Identification and gating of Synechoccocus followed a protocol by Marie et al. (1997).

☒ Tick this box to confirm that a figure exemplifying the gating strategy is provided in the Supplementary Information.
